# Nutritional and inflammatory measures predict survival of patients with stage IV colorectal cancer

**DOI:** 10.1186/s12885-020-07560-3

**Published:** 2020-11-11

**Authors:** Yasuyuki Takamizawa, Dai Shida, Narikazu Boku, Yuya Nakamura, Yuka Ahiko, Takefumi Yoshida, Taro Tanabe, Atsuo Takashima, Yukihide Kanemitsu

**Affiliations:** 1 Department of Colorectal Surgery, National Cancer Center Hospital, 5-1-1 Tsukiji, Chuo-ku, Tokyo, 1040045 Japan; 2 Division of Frontier Surgery, The Institute of Medical Science, The University of Tokyo, 4-6-1 Shirokanedai, Minato-ku, Tokyo, 1088639 Japan; 3 Gastrointestinal Medical Oncology Division, National Cancer Center Hospital, 5-1-1 Tsukiji, Chuo-ku, Tokyo, 1040045 Japan

**Keywords:** Controlling nutritional status (CONUT) score, Colorectal cancer, Modified Glasgow prognostic score (mGPS), Prognostic nutritional index (PNI)

## Abstract

**Background:**

This study aimed to evaluate the prognostic impact of nutritional and inflammatory measures (controlling nutritional status (CONUT) score, prognostic nutritional index (PNI), and modified Glasgow prognostic score (mGPS)) on overall survival (OS) in patients with stage IV colorectal cancer (CRC).

**Methods:**

Subjects were 996 patients with stage IV CRC who were referred to the National Cancer Center Hospital between 2001 and 2015. We retrospectively investigated correlations between OS and CONUT score, PNI, and mGPS. Multivariate analyses were performed using Cox proportional hazards regression models.

**Results:**

After adjusting for known factors (age, gender, BMI, ECOG performance status, location of primary tumor, CEA levels, histological type, M category, and prior surgical treatment), all three measures were found to be independent prognostic factors for OS in patients with stage (CONUT score, *p* < 0.001; PNI, *p* < 0.001; mGPS, *p* < 0.001). Significant differences in OS were found between low CONUT score (0/1) (*n* = 614; 61%) and intermediate CONUT score (2/3) (*n* = 276; 28%) (hazard ratio (HR) = 1.20, 95% confidence interval (CI): 1.02–1.42, *p* = 0.032), and intermediate CONUT score and high CONUT score (≥4) (*n* = 106; 11%) (HR = 1.30, 95% CI: 1.01–1.67, *p* = 0.045). Significant differences in OS were found between mGPS = 0 (*n* = 633; 64%) and mGPS = 1 (*n* = 234; 23%) (HR = 1.84, 95% CI: 1.54–2.19, *p* < 0.001), but not between mGPS = 1 and mGPS = 2 (*n* = 129; 13%) (HR = 1.12, 95% CI: 0.88–1.41, *p* = 0.349). Patients with low PNI (< 48.0) (*n* = 443; 44%) showed a significantly lower OS rate than those with high PNI (≥48.0) (*n* = 553; 56%) (HR = 1.39, 95% CI: 1.19–1.62, *p* < 0.001).

**Conclusions:**

CONUT score, PNI, and mGPS were found to be independent prognostic factors for OS in patients with stage IV CRC, suggesting that nutritional and inflammatory status is a useful host-related prognostic indicator in stage IV CRC.

## Background

The tumor-node-metastasis (TNM) eighth edition introduces a new structure, referred to as the ‘prognostic factors grid,’ which consists of prognostic factors for survival in various cancers [1]. Although the anatomical extent of disease as categorized by TNM is a very powerful prognostic indicator in cancer, ‘host-related (patient profile)’ factors are also considered to have a significant impact on survival. Reportedly, modified Glasgow prognostic score (mGPS) and neutrophil-to-lymphocyte ratio (NLR) are both host-related prognostic factors of pancreatic cancer, and ‘nutritional status’ is a host-related prognostic factor of esophageal cancer [1]. Thus, nutritional and inflammatory status has gained attention from the perspective of prognosis in various malignancies [2–4]. On the other hand, only ‘age’ and ‘race,’ but not nutritional and inflammatory status, are included in the host-related prognostic factors of colorectal cancer (CRC) according to the ‘prognostic factors grid’ [1].

mGPS and prognostic nutritional index (PNI) are representative measures of nutritional and inflammatory status. mGPS is calculated from C-reactive protein (CRP) and serum albumin, a common marker of malnutrition and inflammation [5], and PNI is calculated from serum albumin and total peripheral lymphocyte count [6]. Both measures have been reported to be associated with survival in various malignancies including CRC [5–9]. In addition to these two measures, controlling nutritional status (CONUT) score, which is calculated from serum albumin, total peripheral lymphocyte count, and total cholesterol, has recently gained attention as a tool to evaluate the general condition of patients with cancer from nutritional and immunological perspectives [10–12]. CONUT score was originally developed as an easily calculable score to replace Subjective Global Assessment and Full Nutritional Assessment [13]. According to previous studies, CONUT score is a potential prognostic factor for survival in patients with stage I/II/III CRC and metastatic CRC receiving chemotherapy [14–17]. However, prognostic factors for stage IV CRC, which includes curative resected stage IV CRC, have not been adequately examined yet. Proper evaluation of the general condition of patients with stage IV CRC is important to optimize multimodal strategies [18, 19].

In order to determine the optimal treatment strategy for stage IV CRC from the perspective of host status, it is of interest to evaluate whether nutritional and inflammatory measures are prognostic factors for stage IV CRC including curative resected stage IV CRC and metastatic CRC. Accordingly, the present study aimed to evaluate the prognostic impact of three nutritional and inflammatory measures (mGPS, PNI, and CONUT score) on overall survival (OS) in patients with stage IV CRC.

## Methods

### Study population

Subjects were consecutive patients who were initially diagnosed with stage IV CRC, and who were referred to the Department of Colorectal Surgery or Department of Gastrointestinal Oncology at the National Cancer Center Hospital between January 2001 and December 2015. Eligible patients were required to have stage IV CRC with histologically confirmed adenocarcinoma. Other histological types were excluded. Also excluded were patients with anal cancer or appendiceal cancer, and those with missing data. Initial treatment was determined at multidisciplinary team conferences held by colorectal surgeons, medical oncologists, hepatobiliary surgeons, respiratory surgeons, pathologists, radiologists, and nurses. This retrospective study was approved by the Institutional Review Board (IRB) of the National Cancer Center Hospital (IRB code: 2015–320).

### Data collection

The following parameters were obtained from medical records: age, treatment year, gender, body mass index (BMI), Eastern Cooperative Oncology Group (ECOG) performance status, location of primary tumor (right-sided: the cecum, ascending colon, hepatic flexure, and transverse colon; left-sided: the splenic flexure, descending colon, sigmoid, rectosigmoid junction, and rectum), histological type (‘differentiated,’ defined as tubular adenocarcinoma and papillary adenocarcinoma; ‘others,’ defined as poorly differentiated adenocarcinoma, mucinous adenocarcinoma, and signet-ring cell carcinoma), and pretreatment serum carcinoembryonic antigen (CEA) levels. The M category was assessed according to the Union for International Cancer Control TNM classification (eighth edition), which was recently revised to include the following three subcategories: M1a (metastasis confined to one organ), M1b (metastasis to more than one organ), and M1c (metastasis to the peritoneum with or without other organ involvement) [1]. As for the curability of surgical treatment, subjects were classified into the following two groups: patients who received curative resection of both primary tumor and metastatic lesions, and patients who received no curative resection including those who received palliative primary tumor.

Blood samples were obtained at the time of first visit or before initial treatment. CONUT scores were calculated using serum albumin, total peripheral lymphocyte count, and total cholesterol based on a previous report [13]. Albumin concentrations ≥3.5, 3.0–3.49, 2.5–2.99, and < 2.5 g/dL were scored as 0, 2, 4, and 6 points, respectively; total lymphocyte counts ≥1600, 1200–1599, 800–1199, and < 800/mm^3^ were scored as 0, 1, 2, and 3 points, respectively; and total cholesterol concentrations ≥180, 140–179, 100–139, and < 100 mg/ dL were scored as 0, 1, 2, and 3 points, respectively. PNI was calculated as 10 × albumin concentration (g/dl) + 0.005 × total lymphocyte count (/mm^3^) [6]. mGPS was scored as follows: score 0, CRP ≤1.0 mg/dL; score 1, CRP > 1.0 mg/dL and albumin ≥3.5 g/dL; and score 2, CRP > 1.0 mg/dL and albumin < 3.5 g/dL [5].

Cut-off values of CONUT score, PNI, and mGPS were determined as follows. For CONUT score, which is a categorical variable (ordinal variable) in the range of 0 to 12, cut-off values vary by study as patients are generally classified into two to four groups according to scores [14, 20]. In the present study, patients were divided into the following three groups based on pretreatment data: low (0/1), intermediate (2/3), and high (≥4). For PNI, a numerical variable (continuous variable), a receiver operating characteristic (ROC) curve analysis was performed with survival at 2 years from diagnosis as the outcome, taking into consideration that MST for unresectable colorectal cancer is roughly 30 months. For mGPS, a categorical variable (ordinal variable), analysis was performed by dividing patients into three groups (0, 1, and 2).

### Treatment and follow-up

For follow-up after curative resection, serum tumor marker measurements were performed every one to 3 months, and computed tomography (CT) scans were performed every three to 6 months, with a cut-off date of July 2019, as described previously [21, 22]. According to Japanese Society for Cancer of the Colon and Rectum (JSCCR) guidelines 2016 [23], postoperative chemotherapy after curative resection was not usually performed. Patients with initially unresectable stage IV CRC underwent systemic chemotherapy (multiple cytotoxic agent therapy with or without molecular targeted agents) continuously with or without palliative resection of primary tumor.

### Statistical analysis

Pearson’s chi-square test was used for categorical variables, and the Wilcoxon rank-sum test was used for continuous variables. OS was defined as the interval between the date of stage IV CRC diagnosis and the date of all-cause death. Survival rates were calculated by the Kaplan-Meier method, and survival curves were compared with the log-rank test. Survivors were censored as of the date of data cut-off (July 2019). Multivariate analyses were performed using Cox proportional hazards regression models to evaluate the prognostic impact of each factor on OS. The following known factors were included: age [1], gender, BMI, ECOG performance status, location of primary tumor [24], CEA levels [21], histological type, M category [22], and surgical treatment, as well as CONUT score, PNI, and mGPS, given the overlap in elements constituting each measure.

Data are expressed as numbers of patients, ratios (%), or hazard ratios (HRs) and 95% confidence intervals (CIs), as indicated. Differences with a *P*-value < 0.05 were considered statistically significant. All analyses were performed using JMP14 software (SAS Institute Japan Ltd., Tokyo, Japan).

## Results

### Study population

**Fig. 1:**
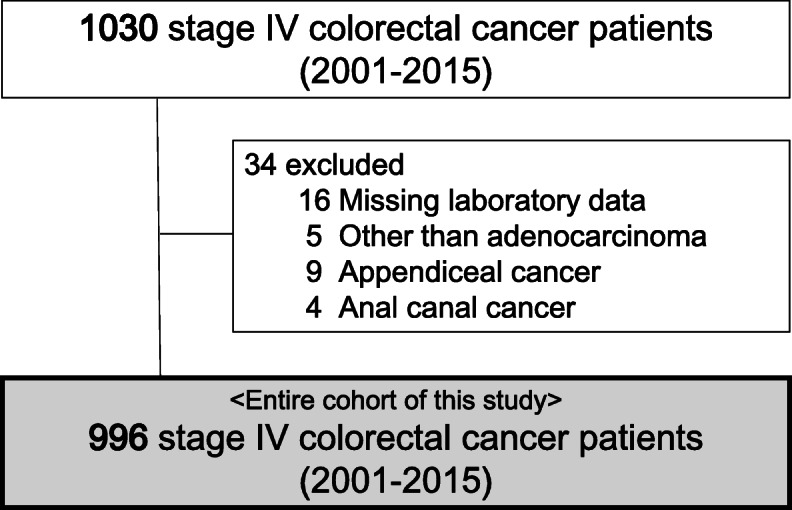
Patient flow diagram. The final study cohort consisted of 996 patients with stage IV colorectal cancer

A total of 1030 patients initially diagnosed with stage IV CRC were identified. Of these, patients with missing laboratory data (*n* = 16), patients with histologic diagnoses other than adenocarcinoma (e.g., neuroendocrine tumor) (*n* = 5), and patients with appendiceal cancer (*n* = 9) or anal canal cancer (*n* = 4) were excluded. Therefore, the study cohort consisted of 996 patients with stage IV CRC (Fig. 1). Follow-up was conducted for the entire cohort, with a median follow-up time of 53 months (range, 1–228 months) among survivors.

### Patient characteristics

Relationships between clinicopathological characteristics and CONUT score, PNI, and mGPS are summarized in Table 1. Among the 996 CRC patients included in this study, 573 (58%) were male and 423 (42%) were female, with a median age of 61.0 years (range, 20–91 years). The median PNI was 48.5 (range, 24.5–68.0). According to ROC curve analysis of death within 2 years after diagnosis, 48.0 was set as the cut-off value of PNI (sensitivity: 57.3%, specificity: 64.7%). The median CONUT score was 1 (range, 0–11) and the median mGPS was 0 (range, 0–2). Almost two thirds of patients were categorized into the low-CONUT group (0/1), whereas 28 and 11% were categorized into the intermediate-CONUT group (2/3) and high-CONUT group (≥4), respectively. Statistical analyses revealed significant associations between high CONUT scores and low BMI (< 20 kg/m^2^) (*p* < 0.001), worse ECOG performance status (PS2, PS3, PS4) (*p* < 0.001), right-sided primary tumor (*p* = 0.004), high CEA levels (≥30 ng/ml) (*p* < 0.001), and low rate of curative resection (*p* < 0.001). Significant associations were also observed between low PNI and older age (≥65 years) (*p* < 0.001), female (*p* = 0.041), low BMI (*p* < 0.001), worse ECOG performance status (*p* = 0.001), right-sided primary tumor (*p* < 0.001), high CEA levels (*p* < 0.001), and low rate of curative resection (*p* < 0.001), and between mGPS = 2 and low BMI (*p* < 0.001), worse ECOG performance status (*p* < 0.001), right-sided primary tumor (*p* = 0.035), high CEA levels (*p* < 0.001), low rate of M1a (*p* < 0.001), and low rate of curative resection *(p* < 0.001).

**Table 1:** Relationships between clinicopathological characteristics and CONUT score, PNI, and mGPS

			CONUT score							PNI					mGPS						
	All cases		Low (0/1)		Intermediate (2/3)		High (≥4)		***P*** value	Low (< 48.0)		High (≥48.0)		***P*** value	0		1		2		***P*** value
Cases	996		614 (61%)		276 (28%)		106 (11%)			443 (44%)		553 (56%)			633 (64%)		234 (23%)		129 (13%)		
Age (years)																					
< 65	625 (63%)		399 (65%)		170 (62%)		56 (53%)		0.052	249 (56%)		376 (68%)		< 0.001	392 (62%)		160 (68%)		73 (57%)		0.066
≥ 65	371 (37%)		215 (35%)		106 (38%)		50 (47%)			194 (44%)		177 (32%)			241 (38%)		74 (32%)		56 (43%)		
Gender																					
Male	573 (58%)		340 (55%)		167 (61%)		66 (62%)		0.208	239 (54%)		334 (60%)		0.041	277 (44%)		93 (40%)		53 (41%)		0.537
Female	423 (42%)		274 (45%)		109 (39%)		40 (38%)			204 (46%)		219 (40%)			356 (56%)		141 (60%)		76 (59%)		
BMI																					
< 20 kg/m^2^	264 (27%)		131 (21%)		89 (32%)		44 (42%)		< 0.001	152 (34%)		112 (20%)		< 0.001	162 (26%)		51 (22%)		51 (40%)		< 0.001
≥ 20 kg/m^2^	732 (73%)		483 (79%)		187 (68%)		62 (58%)			291 (66%)		441 (80%)			471 (74%)		183 (78%)		78 (60%)		
ECOG performance status																					
PS0, PS1	961 (96%)		600 (98%)		267 (97%)		94 (89%)		< 0.001	418 (94%)		543 (98%)		0.001	623 (98%)		223 (95%)		115 (89%)		< 0.001
PS2, PS3, PS4	35 (4%)		14 (2%)		9 (3%)		12 (11%)			25 (6%)		10 (2%)			10 (2%)		11 (5%)		14 (11%)		
Location of primary tumor																					
Right-sided	293 (29%)		158 (26%)		95 (34%)		40 (38%)		0.004	163 (37%)		130 (24%)		< 0.001	169 (27%)		77 (33%)		47 (36%)		0.035
Left-sided	703 (71%)		456 (74%)		181 (66%)		66 (62%)			280 (63%)		423 (76%)			464 (73%)		157 (67%)		82 (64%)		
CEA																					
< 30 ng/ml	524 (53%)		351 (57%)		133 (48%)		40 (38%)		< 0.001	186 (42%)		338 (61%)		< 0.001	404 (64%)		86 (37%)		34 (26%)		< 0.001
≥ 30 ng/ml	472 (47%)		263 (43%)		143 (52%)		66 (62%)			257 (58%)		215 (39%)			229 (36%)		148 (63%)		95 (74%)		
Histological type																					
Differentiated	897 (90%)		553 (90%)		249 (90%)		95 (90%)		0.985	396 (89%)		501 (91%)		0.527	576 (91%)		205 (88%)		116 (90%)		0.334
Others	99 (10%)		61 (10%)		27 (10%)		11 (10%)			47 (11%)		52 (9%)			57 (9%)		29 (12%)		13 (10%)		
M category																					
M1a	553 (56%)		355 (58%)		145 (53%)		53 (50%)		0.268	230 (52%)		323 (58%)		0.080	388 (61%)		106 (45%)		59 (46%)		< 0.001
M1b	227 (23%)		132 (21%)		64 (23%)		31 (29%)			114 (26%)		113 (20%)			119 (19%)		64 (27%)		44 (34%)		
M1c	216 (22%)		127 (21%)		67 (24%)		22 (21%)			99 (22%)		117 (21%)			126 (20%)		64 (27%)		26 (20%)		
Surgical treatment																					
Curative resection	302 (30%)		208 (34%)		74 (27%)		20 (19%)		< 0.001	101 (23%)		201 (36%)		< 0.001	257 (40%)		33 (14%)		12 (9%)		< 0.001
Palliative resection of primary tumor	303 (30%)	694 (70%)	208 (34%)	406 (66%)	74 (27%)	202 (73%)	21 (20%)	86 (81%)		120 (27%)	342 (77%)	183 (33%)	352 (64%)		207 (33%)	376 (60%)	68 (29%)	201 (86%)	28 (22%)	117 (91%)	
No resection	391 (40%)		198 (32%)		128 (46%)		65 (61%)			222 (50%)		169 (31%)			169 (27%)		133 (57%)		89 (69%)		
Albumin (g/dl)	4.0 (3.7–4.3)		4.1 (3.9–4.4)		3.9 (3.5–4.2)		3.1 (2.8–3.4)		< 0001	3.7 (3.3–3.9)		4.3 (4.1–4.4)		< 0.001	4.2 (4.0–4.4)		3.8 (3.7–4.0)		3.2 (2.9–3.3)		< 0.001
CRP (mg/dl)	0.5 (0.1–2.0)		0.3 (0.1–1.1)		0.8 (0.2–2.7)		4.3 (1.2–8.2)		< 0001	1.5 (0.4–4.6)		0.2 (0.1–0.7)		< 0.001	0.2 (0.1–0.4)		2.3 (1.4–4.1)		5.8 (2.6–8.4)		< 0.001
Total cholesterol (mg/dl)	203 (173–240)		210 (186–243)		193 (165–235)		162 (136–221)		< 0001	196 (164–239)		208 (182–240)		< 0.001	203 (176–233)		201 (172–257)		205 (157–256)		0.574
Lymphocytes (mm^3^)	1600 (1200–2000)		1800 (1520–2200)		1125 (1000–1500)		1105 (900–1508)		< 0001	1300 (1070–1600)		1870 (1500–2270)		< 0.001	1640 (1300–2000)		1600 (1200–2100)		1320 (1070–1700)		< 0.001

*CONUT* Controlling nutritional status, *PNI* Prognostic nutritional index, *mGPS* Modified Glasgow prognostic score, *BMI* Body mass index, *ECOG PS* Eastern Cooperative Oncology Group performance status, *CEA* Carcinoembryonic antigenNumerical data are expressed as median (25–75% interquartile range)

### Survival

**Fig. 2:**
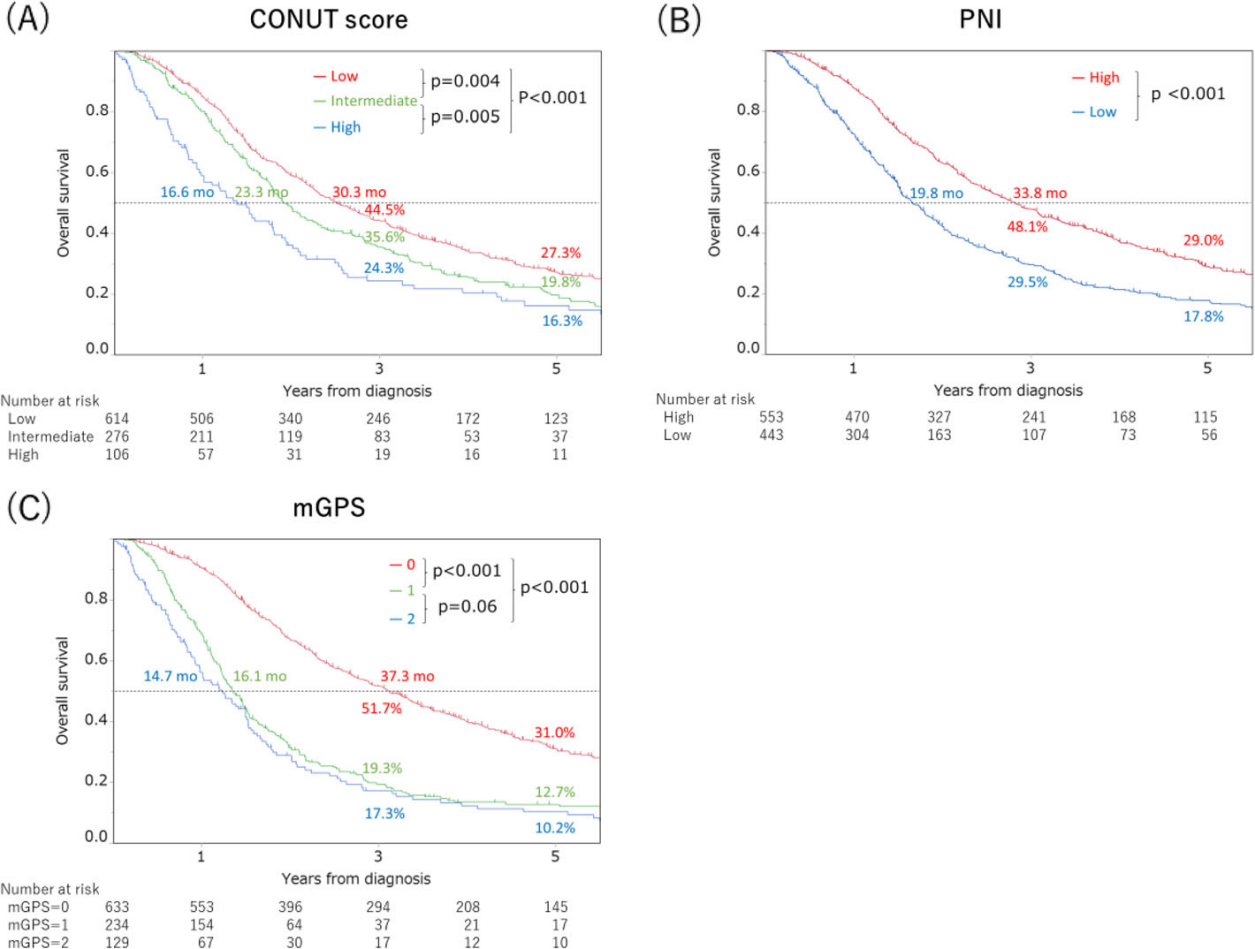
Kaplan-Meier survival curves of overall survival (OS) in patients with stage IV colorectal cancer. Each percentage (%) represent 3- and 5-year OS rates, respectively. Each month (mo) represent median survival time. **a**. Relationship between CONUT score and OS of patients with stage IV colorectal cancer (*P* < 0.001). Patients were divided into three groups according to CONUT score: low (0/1), intermediate (2/3), and high (4 or higher) groups. **b**. Relationship between PNI and OS of patients with stage IV colorectal cancer (*P* < 0.001). According to receiver operating characteristic (ROC) curve analysis, we set 48.0 as the cut-off value (sensitivity: 57.3%, specificity: 64.7%). **c**. Relationship between mGPS and OS of patients with stage IV colorectal cancer (*P* < 0.001)

OS curves according to CONUT score, PNI, and mGPS are provided in Figs. 2a-c. Median survival time was 30.3 months in the low-CONUT group, 23.3 months in the intermediate-CONUT group, and 16.6 months in the high-CONUT group, with 3-year OS rates of 44.5, 35.6, and 24.3%, respectively, and 5-year OS rates of 27.3, 19.8, and 16.3%, respectively (*p* < 0.001). Higher CONUT scores were significantly associated with worse prognoses (Fig. 2a).

Median survival time was 33.8 months in the high-PNI group and 19.8 months in the low-PNI group, with 3-year OS rates of 48.1 and 29.5%, respectively, and 5-year OS rates of 29.0 and 17.8%, respectively (*p* < 0.001). The low-PNI group showed a significantly shorter OS than the high-PNI group (*p* < 0.001) (Fig. 2b).

Median survival time was 37.3 months in the mGPS = 0 group, 16.1 months in the mGPS = 1 group, and 14.7 months in the mGPS = 2 group, with 3-year OS rates of 51.7, 19.3, and 17.3%, respectively, and 5-year OS rates of 31.0, 12.7, and 10.2%, respectively (*p* < 0.001). OS was significantly shorter in mGPS = 2 and mGPS = 1 groups compared to the mGPS = 0 group (*p* < 0.001) (Fig. 2c).

### Clinical factors affecting prognosis

In univariate analysis, CONUT score (*p* < 0.001), PNI (*p* < 0.001), and mGPS *(p* < 0.001), as well as gender (*p* = 0.028), BMI (*p* = 0.008), ECOG performance status (*p* < 0.001), location of primary tumor (*p* < 0.001), CEA levels (*p* < 0.001), histological type (*p* < 0.001), M category (*p* < 0.001), and surgical treatment (*p* < 0.001), were associated with prognosis.

**Fig. 3:**
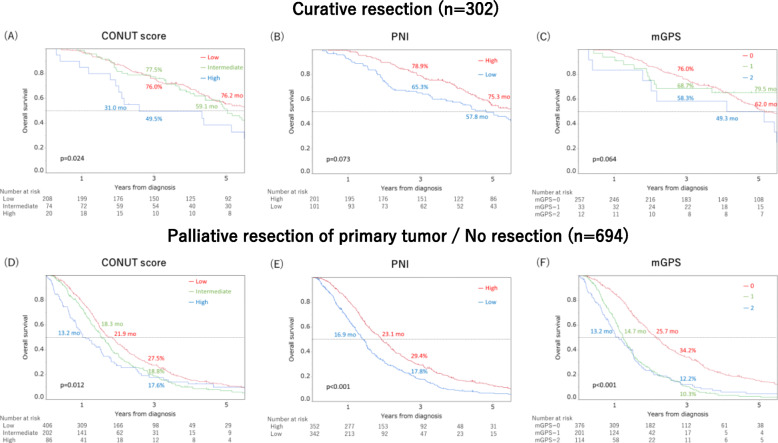
Kaplan-Meier survival curves of overall survival (OS) in patients with stage IV colorectal cancer. Each percentage (%) represent 3-year OS rates. Each month (mo) represent median survival time. **a**-**c**. Relationships between three nutritional and inflammatory measures (**a**: CONUT score, *p* = 0.024, **b**: PNI, *p* = 0.073, **c**: mGPS, *p* = 0.064) and OS of patients with stage IV colorectal cancer who received curative resection (*n* = 302). **d**-**f**. Relationships between three nutritional and inflammatory measures (**d**: CONUT score, *p* = 0.012, **e**: PNI, *p* < 0.001, **f**: mGPS, *p* < 0.001) and OS of patients with stage IV colorectal cancer who received palliative resection of primary tumor or no resection (*n* = 694)

Subgroup analyses were performed by dividing the patients into those who underwent curative resection (*n* = 302) and those who underwent palliative resection of primary tumor / no resection (*n* = 694). Kaplan-Meier survival curves comparing OS between the two subgroups according to the three nutritional and inflammatory measures are shown in Figs. 3a-f. OS curves for both subgroups showed similar trends to the overall results (Curative resection: CONUT score, *p* = 0.024; PNI, *p* = 0.073; mGPS, *p* = 0.064; Palliative resection of primary tumor / no resection: CONUT score, *p* = 0.012; PNI, *p* < 0.001; mGPS, *p* < 0.001).

Multivariate analyses were performed, adjusting for clinical factors that were significant in univariate analyses (gender, BMI, ECOG performance status, location of primary tumor, CEA levels, histological type, M category, and surgical treatment); ‘age’ was also included given the prior knowledge according to TNM eighth edition [1]. All three measures were found to be independent prognostic factors for OS in patients with stage IV CRC (CONUT score, *p* < 0.001; PNI, *p* < 0.001; mGPS, *p* < 0.001). Significant differences in OS were found between the low-CONUT group and intermediate-CONUT group (HR = 1.20, 95% CI: 1.02–1.42, *p* = 0.032), low-CONUT group and high-CONUT group (HR = 1.57, 95% CI: 1.23–1.98, *p* < 0.001), and intermediate-CONUT group and high-CONUT group (HR = 1.30, 95% CI: 1.01–1.67, *p* = 0.045). In contrast, for mGPS, significant differences in OS were found between mGPS = 0 and mGPS = 1 groups (HR = 1.84, 95% CI: 1.54–2.19, *p* < 0.001) and mGPS = 0 and mGPS = 2 groups (HR = 2.06, 95% CI: 1.65–2.55, *p* < 0.001), but not between mGPS = 1 and mGPS = 2 groups (HR = 1.12, 95% CI: 0.88–1.41, *p* = 0.349). For PNI, the low-PNI group had a significantly lower OS rate than the high-PNI group (HR = 1.39, 95% CI: 1.19–1.62, *p* < 0.001) (Table 2).

**Table 2:** Univariate and Multivariate Analyses for OS

Variable	Category	Reference	Univariate		Multivariate^**a**^	
			HR (95% CI)	***p*** value	HR (95% CI)	***p*** value
CONUT score	Intermediate (2/3)	Low (0/1)	1.27 (1.08–1.49)	0.004	1.20 (1.02–1.42)	0.032
	High (≥4)	Low (0/1)	1.84 (1.45–2.31)	< 0.001	1.57 (1.23–1.98)	< 0.001
	High (≥4)	Intermediate (2/3)	1.45 (1.13–1.86)	0.005	1.30 (1.01–1.67)	0.045
PNI	Low (< 48.0)	High (≥48.0)	1.62 (1.40–1.87)	< 0.001	1.39 (1.19–1.62)	< 0.001
mGPS	1	0	2.26 (1.90–2.67)	< 0.001	1.84 (1.54–2.19)	< 0.001
	2	0	2.83 (2.28–3.47)	< 0.001	2.06 (1.65–2.55)	< 0.001
	2	1	1.25 (0.99–1.58)	0.063	1.12 (0.88–1.41)	0.349

^a^ Adjusted for the following variables: age, gender, BMI, ECOG performance status,

location of primary tumor, CEA levels, histological type, M category, and surgical treatment

Data are presented as hazard ratios and 95% confidence intervals

*HR* Hazard ratio, *CI* Confidence interval, *CONUT* Controlling nutritional status

*PNI* Prognostic nutritional index, *mGPS* Modified Glasgow prognostic score

## Discussion

In the present study, we focused on stage IV CRC including curative resected stage IV CRC and unresectable metastatic CRC, and demonstrated that CONUT score, PNI, and mGPS are independent prognostic factors for OS in patients with stage IV CRC regardless of curative potential. Our results are compatible with previous reports that PNI, mGPS, and CONUT score were prognostic factors in CRC patients undergoing curative resection [5, 7, 8, 14, 17], and that CONUT score was a prognostic factor in metastatic CRC patients undergoing chemotherapy [16]. To our knowledge, this is the largest study to date that comprehensively assessed the prognostic significance of nutritional and inflammatory measures in patients with stage IV CRC. Given that nutritional and/or inflammatory status has been recognized as a host-related prognostic factor in pancreatic cancer and esophageal cancer [1], nutritional and inflammatory measures may also be useful for stage IV CRC in daily clinical practice.

We demonstrated that three nutritional and inflammatory measures tended to stratify OS in subgroup analysis according to the treatment strategy. Furthermore, multivariate analyses adjusted for known factors including surgical treatment revealed that the three nutritional and inflammatory measures were all independent prognostic factors for OS in patients with stage IV CRC. These results suggest that nutritional and inflammatory measures may be a useful prognostic indicator regardless of treatment strategies. It is acceptable because nutritional status affects tolerability not only surgery but also chemotherapy [2, 25–29]. Patients with advanced cancer are prone to malnutrition, which in turn can lead to postoperative complications and worse postoperative survival [25, 28, 29]. Malnutrition is also associated with severe chemotherapy-related toxicity and reduced survival [2, 27]. Although treatment strategies for stage IV CRC vary depending on guidelines [23, 30, 31], nutritional and inflammatory measures can be applied to the entire population of stage IV CRC patients.

The three nutritional and inflammatory measures evaluated in this study are calculated based on serum albumin, which serves as a valuable predictor of nutritional status and disease severity in chronically and critically ill patients. Cancer-related proinflammatory cytokines, such as interleukin-6 (IL-6) and interleukin-1 (IL-1), as well as tumor necrosis factor-α (TNF-α) inhibit albumin production, leading to cancer cachexia [32]. Serum albumin is also an independent prognostic indicator in several cancers [33]. Other components of CONUT score, PNI, and mGPS (i.e., CRP, lymphocytes, cholesterol) are also markers of cancer-induced inflammation or immunity to cancer. CRP, a protein synthesized in hepatocytes, belongs to a family of acute phase proteins and is regulated by cytokines such as IL-6 and TNF-α [34]. CRP has been reported to be associated with both the malignant potential of neoplasms and physical cachexia, and several studies have shown that cancer patients with elevated serum CRP levels had a worse prognosis than those without [35]. Lymphocytes play an important role in immune response to cancer [36]. Cholesterol is a fundamental component of cellular membranes involved in the cellular signaling pathway, which plays an essential role in cell growth and differentiation [37]. Low serum cholesterol levels indicate a lack of caloric intake and impairment in the immune system. Although the relationship between serum cholesterol and cancer remains controversial, several studies have shown that low serum cholesterol is associated with an increased risk of cancer and worse prognosis in cancer [38, 39]. The present study revealed that CONUT score, PNI, and mGPS, which consist of these factors, are strongly correlated with prognosis in stage IV CRC patients.

Many nutritional and inflammatory measures have been reported to be associated with CRC prognosis. mGPS, or GPS, is a representative and evidence-based marker in CRC. GPS is considered a useful predictor of postoperative mortality in patients with CRC [7], as well as for OS after surgery in stage IV CRC [40]. PNI is a useful predictor for OS of patients with stage IV CRC who underwent palliative resection [28]. In a meta-analysis of CRC patients who underwent primary tumor resection (curative-intent resection), PNI was found to be a prognostic indicator of postoperative OS [41]. CONUT score is a relatively new measure reported to be useful for predicting postoperative OS in early-stage CRC [14, 15, 17] and OS of patients with metastatic CRC receiving chemotherapy [16]. In addition to these measures, NLR, platelet-to-lymphocyte ratio (PLR), and lymphocyte-to-monocyte ratio (LMR) have also been reported to be associated with CRC prognosis [42–44].

However, it remains controversial as to which one of those measures is the most useful. Several studies have compared CONUT score with other measures. For instance, Toyokawa et al. [11] reported that CONUT score was an independent predictor of OS and relapse-free survival among thoracic esophageal squamous cell carcinoma patients, and was superior to PLR, NLR, and GPS. Liu et al. [12] reported that CONUT score was an independent prognostic factor in patients with stage II-III gastric cancer receiving curative resection and adjuvant chemotherapy. In the low PNI group, CONUT score effectively stratified cancer-specific survival, suggesting it is potentially a better survival predictor than PNI. The present study focused on three measures calculated from serum albumin to assess patient prognosis in terms of nutritional status as well as inflammation. CONUT score, PNI, and mGPS were adjusted for known factors (i.e., age, gender, BMI, ECOG performance status, location of primary tumor, CEA levels, histological type, M category, and surgical treatment), and all three measures were independent prognostic factors in patients with stage IV CRC. Among them, however, CONUT score was significantly associated with OS. In contrast, there was no significant difference in OS between consecutive mGPS scores, suggesting that the distribution of CONUT scores was more balanced than that of mGPS scores. Unlike CONUT score and mGPS, there were only two groups by PNI; thus, CONUT scores may be more useful in stratifying patients with stage IV CRC compared to PNI and mGPS.

Currently, there exists no established view on what interventions should be used for patients who are identified as having a poor prognosis according to these nutritional and inflammatory measures. It also remains unclear whether nutritional and inflammatory status changes during the clinical progression of the disease. While the impact of nutritional interventions on nutritional and inflammatory measures is unknown, nutritional interventions have been shown to improve clinical outcomes of surgery and chemotherapy [45–48]. Nutritional interventions should thus be considered for malnourished patients.

It is worth noting that the three nutritional and inflammatory measures evaluated in this study do not meet the GLIM criteria, global consensus on the definition of malnutrition. According to the GLIM criteria, malnutrition is defined as meeting one of three phenotypic criteria (non-volitional weight loss, low BMI, reduced muscle mass) and one of two etiological criteria (reduced food intake or assimilation, disease burden/inflammation) [49]. CONUT score, PNI, and mGPS are supportive tools for assessing the nutritional status of the host in terms of inflammation. Since these measures cannot be used to fully assess the nutritional status of patients, appropriate nutritional assessments should be performed, and nutritional interventions offered, to those who are identified as having a poor prognosis/malnutrition according to these measures. Further prospective studies are warranted to assess how nutritional interventions can improve nutritional and inflammatory measures.

This study has some limitations. First, given the retrospective design and collection of data from one institution, there may have been selection bias. Second, although nutritional management and use of supplements or therapeutic diets are important confounding factors in assessing the usefulness of nutritional and inflammatory measures in predicting cancer prognosis, we did not have these information. Third, although consecutive patients were enrolled, there have been significant changes during the long study period (2001 to 2015) in treatment strategies, such as chemotherapy. Thus, our study may not be fully reflective of current medical practice. The correlation between nutritional and inflammatory status and prognosis in stage IV CRC warrants further consideration and validation in prospective studies.

## Conclusions

CONUT score, PNI, and mGPS were all independent prognostic factors for OS in patients with stage IV CRC, suggesting that nutritional and inflammatory status is a useful host-related prognostic indicator in stage IV CRC. Regardless of the TNM stage, the use of nutritional and inflammatory measures should be considered in daily clinical practice to assess host status.

## Data Availability

The datasets used and/or analyzed during the current study are available from the corresponding author on reasonable request.

## Abbreviations

95% CI: 95% Confidence interval
CEA: Carcinoembryonic antigen,
CONUT: Controlling nutritional status
CRC: Colorectal cancer
ECOG: Eastern cooperative oncology group
HR: Hazard ratio
IL-1: Interleukin-1
IL-6: Interleukin-6
LMR: Lymphocyte-to-monocyte ratio
NLR: Neutrophil-to-lymphocyte ratio
mGPS: Modified glasgow prognostic score
OS: Overall survival
PLR: Platelet-to-lymphocyte ratio
PNI: Prognostic nutritional index
TNF-α: Tumor necrosis factor-α
TNM: Tumor-node-metastasis
